# Timing of pubertal stages and breast cancer risk: the Breakthrough Generations Study

**DOI:** 10.1186/bcr3613

**Published:** 2014-02-04

**Authors:** Danielle H Bodicoat, Minouk J Schoemaker, Michael E Jones, Emily McFadden, James Griffin, Alan Ashworth, Anthony J Swerdlow

**Affiliations:** 1 Division of Genetics and Epidemiology, Institute of Cancer Research, Sutton, UK; 2 Diabetes Research Centre, University of Leicester, Leicester, UK; 3 Department of Primary Care Health Sciences, Oxford University, Oxford, UK; 4 Breakthrough Breast Cancer Research Centre and Division of Molecular Pathology, Institute of Cancer Research, London, UK; 5 Division of Breast Cancer Research, Institute of Cancer Research, Sutton, UK; 6 Diabetes Research Centre, University of Leicester, Leicester Diabetes Centre, Leicester General Hospital, Gwendolen Road, Leicester, Leicestershire LE5 4PW, UK

## Abstract

**Introduction:**

Breast development and hormonal changes at puberty might affect breast cancer risk, but epidemiological analyses have focussed largely on age at menarche and not at other pubertal stages.

**Methods:**

We investigated associations between the timing of pubertal stages and breast cancer risk using data from a cohort study of 104,931 women (Breakthrough Generations Study, UK, 2003–2013). Pubertal variables were reported retrospectively at baseline. Breast cancer risk was analysed using Cox regression models with breast cancer diagnosis as the outcome of interest, attained age as the underlying time variable, and adjustment for potentially confounding variables.

**Results:**

During follow-up (mean = 4.1 years), 1094 breast cancers (including ductal carcinoma *in situ*) occurred. An increased breast cancer risk was associated with earlier thelarche (age when breast growth begins; HR [95% CI] = 1.23 [1.02, 1.48], 1 [referent] and 0.80 [0.69, 0.93] for ≤10, 11–12 and ≥13 years respectively), menarche (initiation of menses; 1.06 [0.93, 1.21], 1 [referent] and 0.78 [0.62, 0.99] for ≤12, 13–14 and ≥15 years), regular periods (0.99 [0.83, 1.18], 1 [referent] and 0.74 [0.59, 0.92] for ≤12, 13–14 and ≥15 years) and age reached adult height (1.25 [1.03, 1.52], 1 [referent] and 1.07 [0.87, 1.32] for ≤14, 15–16 and ≥17 years), and with increased time between thelarche and menarche (0.87 [0.65, 1.15], 1 [referent], 1.14 [0.96, 1.34] and 1.27 [1.04, 1.55] for <0, 0, 1 and ≥2 years), and shorter time between menarche and regular periods (1 [referent], 0.87 [0.73, 1.04] and 0.66 [0.50, 0.88] for 0, 1 and ≥2 years). These associations were generally similar when considered separately for premenopausal and postmenopausal breast cancer.

**Conclusions:**

Breast duct development may be a time of heightened susceptibility to risk of carcinogenesis, and greater attention needs to be given to the relation of breast cancer risk to the different stages of puberty.

## Introduction

Breast cancer is the most common invasive cancer among women world-wide [1]. Timing of puberty is a potentially important risk factor for this tumour, in terms of breast growth and the large hormonal changes that occur around that time. There are several stages of puberty, but epidemiological analyses of breast cancer risk have focussed largely on age at menarche, perhaps because it can be most readily recalled with reasonable accuracy [2]. The stages of puberty, as outlined by Tanner, are thelarche (the start of breast growth), pubarche (the start of pubic hair growth), peak growth (an acceleration of linear growth) and menarche (first menses) [3]. Pubertal phases relevant to breast growth are thelarche, when growth begins, and menarche, when it accelerates [4]; pubertal hormonal surges are detectable by peak growth and the start of menstruation.

Menarcheal age is a well-established breast cancer risk factor [5], with an estimated 10% reduction in breast cancer risk for each two-year increase in age at menarche [6]. Additionally, there is limited evidence indicating higher breast cancer risk in women whose menses become regular sooner, rather than later, after menarche [7,8]. Aside from age at menarche and onset of regular menses, the other Tanner stages have been little studied in relation to breast cancer risk. There is weak evidence that earlier peak growth might increase breast cancer risk [9], but data are minimal, and there appear to have been no studies of risk by age at thelarche.

We, therefore, investigated the associations of age at various pubertal stages and the duration between these stages with breast cancer risk in the Breakthrough Generations Study (BGS), a large cohort study.

## Methods

### Subjects

The BGS is a UK cohort study focussed on breast cancer aetiology. It has received approval from the South Thames Multicentre Research Ethics Committee and all participants gave written informed consent. All women living in the UK and aged 16 years or older were eligible to join the study. Registered supporters of the charity Breakthrough Breast Cancer were invited, and other women joined either by self-referral or by nomination from an existing participant. Over 113,000 participants were recruited between 2003 and 2013. As a requirement of study entry, all BGS participants completed a postal recruitment questionnaire, which included detailed questions about known and possible breast cancer risk factors. Participants are followed-up approximately every two and a half years, using further postal questionnaires that update the recruitment information and reports of cancer diagnoses. Follow-up is based on time since recruitment; thus, at any given time different participants are at different stages of follow-up, because recruitment occurred over 10 years. At present, up to two rounds of follow-up questionnaires have been sent to participants, depending on when they were recruited. Further details of the cohort can be found elsewhere [10].

BGS participants were potentially eligible for the current analyses if they joined the study before 1 April 2010, and thus had reached at least their first follow-up point (*N* = 111,344). Of these, 6,399 women were diagnosed with invasive or *in situ* breast cancer and 14 had a prophylactic bilateral mastectomy before they joined the BGS, and therefore were excluded from the analysis. This left 104,931 subjects who formed the study cohort.

### Variables

Incident invasive and *in situ* breast cancers were identified from participants’ reports in the follow-up questionnaires and spontaneous reports to the study centre, and from flagging at the NHS Central Registers - virtually complete registers of the populations of England and Wales, and of Scotland, on which deaths and cancer registrations are ‘flagged’ and then reported to authorised medical researchers. For a total of 99% (1,082 out of 1,094) of reported cases, the diagnosis was confirmed by cancer registry data or pathology reports (95%) or from the participant’s general practitioner (4%); the remaining 1% were assumed to be true cases because they reported appropriate treatment for breast cancer.

The explanatory and confounding variables analysed were from self-reported data on the recruitment questionnaire. The explanatory variables were age at thelarche, age at menarche, age at regular periods, age when reached adult height, the intervals between these ages, and growth spurt in height between ages 7 and 11 years (as a proxy for peak growth). Age at regular periods was only analysed for women who indicated that their periods had become regular naturally rather than as a consequence of oral contraceptives or some other cause. All ages were reported to the last completed year. Information about height in childhood was collected as self-reported height relative to other girls of the same age, in five categories. Subjects were classed as having had a growth spurt between ages 7 and 11 years if their height compared with girls of a similar age increased by at least one category from age 7 to 11.

The potential confounding variables analysed were age at recruitment (years, continuous), menopausal status (postmenopausal, premenopausal), family history of breast cancer in a first-degree relative (yes, no), adult height (centimetres, continuous), age at first full-term pregnancy (≤24 years, 25 to 29 years, ≥30 years, nulliparous), and hormone replacement therapy (HRT) use (yes, no). These variables were selected as they are breast cancer risk factors and, in exploratory analyses, affected the relationship between at least one of the explanatory variables and breast cancer risk. Adjustments for ethnicity and socio-economic status, (based on place of residence (Acorn scores [11])), were also made, but these had no effect on the results and have not been included in the results presented.

### Statistical analysis

The data were analysed using survival analysis methods. Subjects contributed person-time from recruitment into the BGS until the earliest of: end of follow-up period, diagnosis of invasive or *in situ* breast cancer, death, emigration from the UK or loss to follow-up. The follow-up period was based on the date of the second follow-up questionnaire for the 49,880 women who joined the study before 1 July 2006, and on the date of the first follow-up questionnaire for the remaining women. Participants who were lost to questionnaire follow-up were flagged with the NHS Central Registers, if they had agreed to this. If flagged, they were then censored at 31 December 2010 because national cancer incidence data are currently incomplete after that date; otherwise, they were censored at loss to follow-up.

Cox regression models were fitted in Stata 10.1 (StataCorp, College Station, Texas, USA) [12] with attained age as the underlying time variable, invasive or *in situ* breast cancer diagnosis as the event of interest, and all other outcomes censored. The validity of the proportional hazards assumption was assessed for each model. The initial models were unadjusted but accounted for age, because it was the underlying time variable, and menopausal status, as time at risk was stratified by menopausal status (pre- and post-menopausal). Also analyses were conducted separately for these two menopause categories as pre- and post-menopausal breast cancer have been shown to have different aetiologies to some extent [13]. Menopausal status was based on recruitment questionnaire and subsequently updated from follow-up questionnaires. The date at which a participant became post-menopausal was determined by her reported age at natural menopause or bilateral oophorectomy, or if these were unknown (for example, because of hysterectomy, or hormonal contraceptive or HRT use) at age 50 or the age at which she was last known to be premenopausal, whichever was later. Additional models were fitted that additionally adjusted for family history of breast cancer, adult height, age at first full-term pregnancy and HRT use. These are the results described in the text unless otherwise specified, since both the unadjusted and adjusted findings were very similar. Missing data were not imputed.

Sensitivity analyses were performed by repeating the analyses in various subgroups: women who were younger than age 60 years at recruitment, because recall might be better in these women; after exclusion of women who had a younger first-degree relative in the BGS because of potential correlation within pairs; and taking only invasive or *in situ* breast cancers as the outcome of interest in turn. Receptor status was known for too few women to allow analyses to be split by this factor.

## Results

Table 1 shows descriptive characteristics of the 104,931 BGS participants who formed the cohort analysed. The subjects were aged 16 to 98 years at recruitment (mean = 46.7 years; median = 47 years), and the vast majority were of white ethnicity (98.8%).

**Table 1:** Baseline characteristics of cohort participants

**Variable**	**Mean [SD] or N (%)**
Age at recruitment, years	46.7 [13.4]
Average person-time, years	4.1 [1.7]
Adult height, cm	164.3 [6.6]
Menopausal status	
Postmenopausal	44,373 (42.3)
Premenopausal	60,534 (57.7)
Never periods	24 (0.0)
Family history of breast cancer	
No	89,069 (84.9)
Yes	15,862 (15.1)
Age at first full-term pregnancy, years	
≤24	25,700 (24.5)
25 to 29	31,104 (29.6)
≥30	19,285 (18.4)
Parous; age not reported	166 (0.2)
Nulliparous	28,513 (27.2)
Parity missing	163 (0.2)
Ethnicity	
White	103,655 (98.8)
Mixed white/non-white	585 (0.5)
Non-white	611 (0.6)
Missing	80 (0.1)
Socio-economic status^a^	
1 (Highest)	47,952 (45.7)
2	12,001 (11.4)
3	29,886 (28.5)
4	8,449 (8.0)
5 (Lowest)	6,042 (5.8)
Channel islands/isle of man^b^	497 (0.5)
Unclassified	104 (0.1)
**Total**	**104,931 (100.0)**

SD, Standard deviation.

^a^Socio-economic status was based on placed of residence (Acorn scores).

^b^Residence-based socio-economic status not classifiable.

During follow-up (average follow-up = 4.05 years, total person-years = 424,262), 1,094 (1.0%) women in the cohort were diagnosed with invasive (965; 88.2%) or *in situ* (129; 11.8%) breast cancer, 20 (0.02%) had a prophylactic bilateral mastectomy, 349 (0.3%) died, and 99,545 (94.9%) were followed to the end of their follow-up period. Of the remaining 3,924 (3.7%), 3,240 (3.1%) were followed to December 2010 by flagging, 423 (0.4) were censored at emigration and 261 (0.3%) were lost to follow-up.

Table 2 presents the risk of breast cancer by pubertal factors. An earlier thelarche was associated with higher breast cancer risk; women with a reported age at thelarche ≤10 years had approximately 20% higher risk of breast cancer than those who reported an age at thelarche between 11 and 12 years (HR = 1.23 (95% CI = 1.02, 1.48)). Similarly, thelarche at age 13 years or older was associated with a 20% lower risk of breast cancer (0.80 (0.69, 0.93)). A lower risk of breast cancer was also seen in women whose menarche (0.78 (0.62, 0.99)) or regular periods (0.74 (0.59, 0.92)) onset at 15 years or older compared with age 13 to 14 years, but onset at 12 years or younger was not associated with an increased risk. Conversely, women who reached their adult height at a younger age had an increased risk (1.25 (1.03, 1.52)), but reaching it at an older age (≥17 years) was not protective. Breast cancer risk was not significantly associated with having a linear growth spurt between ages 7 and 11.

**Table 2:** **Risk of invasive or *in situ *** **breast cancer in relation to pubertal variables**

	**Adjusted for attained age**			**Multivariate-adjusted**^**a**^		
**Pubertal variable**	***N *** **cases/person-years**	**HR (95% CI)**		***N *** **cases/person-years**	**HR (95% CI)**	
Age at thelarche, years						
≤10	152/53,916	1.22 (1.02,1.47)	*	151/53,308	1.23 (1.02, 1.48)	*
11 to 12	445/17,5791	Referent		443/173,413	Referent	
≥13	276/120,309	0.82 (0.71,0.96)	*	269/118,581	0.80 (0.69, 0.93)	**
HR for trend		0.82 (0.74, 0.90)	***		0.81 (0.73, 0.89)	***
Age at menarche, years						
≤12	451/171,139	1.03 (0.90,1.17)		449/168,478	1.06 (0.93,1.21)	
13 to 14	449/171,466	Referent		444/168,733	Referent	
≥15	86/38,779	0.81 (0.64,1.02)		83/38,109	0.78 (0.62,0.99)	*
HR for trend		0.92 (0.83, 1.01)			0.89 (0.81, 0.99)	*
Age at regular periods, years						
≤12	213/80,631	0.97 (0.81,1.15)		212/79,450	0.99 (0.83,1.18)	
13 to 14	295/107,008	Referent		292/105,314	Referent	
≥15	105/50,215	0.74 (0.59,0.93)	**	104/49,465	0.74 (0.59,0.92)	**
Never had	73/26,114	1.15 (0.89,1.49)		73/25,581	1.16 (0.90,1.50)	
regular periods						
HR for trend^b^		0.90 (0.81, 1.00)			0.88 (0.79, 0.98)	*
Age reached adult height, years						
≤14	198/73,048	1.20 (0.99,1.45)		198/72,430	1.25 (1.03,1.52)	*
15 to 16	212/100,038	Referent		208/98,651	Referent	
≥17	148/69,134	1.06 (0.86,1.31)		148/67,894	1.07 (0.87,1.32)	
HR for trend		0.93 (0.84, 1.04)			0.92 (0.82, 1.02)	
Growth spurt between ages 7 and 11 years^c^						
No	956/37,8071	Referent		947/372,171	Referent	
Yes	100/34,784	1.15 (0.94,1.41)		98/34,259	1.12 (0.91,1.38)	

CI ,Confidence interval; HR, Hazard ratio.

^*^P <0.05; **P <0.01; ***P <0.001.

^a^Adjusted for attained age, menopausal status, family history of breast cancer in a first-degree relative, adult height, age at first full-term pregnancy (with a term for nulliparous women) and HRT status.

^b^Test for trend excludes those who had never had a regular period.

^c^Subjects were classed as having had a growth spurt between ages 7 and 11 years if their height compared with girls of a similar age increased by at least one category from age 7 to 11.

Table 3 shows the risk of invasive or *in situ* breast cancer by the duration between pubertal events. Breast cancer risk increased as the time between thelarche and menarche increased, but was only significant in those whose menarche occurred at least two years after the onset of thelarche (1.27 (1.04, 1.55)). Breast cancer risk also increased as the time between menarche and regular periods decreased, but again was only significant when the time between the two events was at least two years (0.66 (0.50, 0.88)). Breast cancer risk was not significantly associated with the time between thelarche and onset of regular periods, or between menarche and attaining adult height.

**Table 3:** **Risk of invasive or *in situ *** **breast cancer in relation to duration between pubertal events**

	**Adjusted for attained age**			**Multivariate-adjusted**^**a**^		
**Pubertal variable**	***N *** **cases/person-years**	**HR (95% CI)**		***N *** **cases/person-years**	**HR (95% CI)**	
Thelarche to menarche, years						
<0	57/27,759	0.85 (0.64,1.13)		57/27,381	0.87 (0.65,1.15)	
0	275/112,810	Referent		271/111,168	Referent	
1	303/116,433	1.14 (0.97,1.34)		299/114,967	1.14 (0.96,1.34)	
≥2	156/61,061	1.27 (1.05,1.55)	*	155/60,301	1.27 (1.04,1.55)	*
*P* for trend		1.14 (1.05, 1.23)	**		1.13 (1.05, 1.23)	**
Thelarche to regular periods, years						
<0	24/9,864	0.88 (0.57,1.36)		24/9,734	0.89 (0.58,1.38)	
0	129/46,383	Referent		127/45,750	Referent	
1	179/71,457	0.93 (0.74,1.16)		177/70,595	0.92 (0.73,1.15)	
≥2	188/81,162	0.94 (0.75,1.18)		187/80,149	0.93 (0.74,1.16)	
Never became	73/26,114	1.19 (0.89,1.59)		73/25,581	1.19 (0.89,1.58)	
regular						
*P* for trend^b^		0.99 (0.90, 1.09)			0.98 (0.89, 1.08)	
Menarche to regular periods, years						
0	379/134,744	Referent		376/132,702	Referent	
1	180/71,866	0.88 (0.74,1.06)		178/70,749	0.87 (0.73,1.04)	
≥2	54/31,244	0.67 (0.51,0.90)	**	54/30,778	0.66 (0.50,0.88)	**
Never became	73/26,114	1.14 (0.89,1.47)		73/25,581	1.13 (0.88,1.46)	
regular						
*P* for trend^b^		0.84 (0.75, 0.95)	**		0.84 (0.74, 0.94)	**
Menarche to adult height, years						
<0	14/5,941	0.90 (0.52,1.56)		14/5,871	0.90 (0.52,1.57)	
0 to 1	131/50,937	Referent		130/50,492	Referent	
2-3	191/83,968	0.98 (0.79,1.23)		189/82,911	0.96 (0.77,1.21)	
≥4	165/78,925	0.94 (0.75,1.19)		165/77,560	0.94 (0.75,1.19)	
*P* for trend		0.98 (0.89, 1.09)			0.98 (0.88, 1.09)	

CI, Confidence interval; HR, Hazard ratio.

^*^P <0.05; **P <0.01; *** *P*<0.001.

^a^Adjusted for attained age, menopausal status, family history of breast cancer in a first-degree relative, adult height, age at first full-term pregnancy (with a term for nulliparous women) and HRT status.

^b^Test for trend excludes those who had never had a regular period.

For premenopausal and postmenopausal breast cancer analysed separately (Additional file 1: Tables S1 and S2) and in sensitivity analyses (Additional file 1: Tables S3 and S4), the overall findings were similar to the main results in terms of the effect sizes and general pattern of results. Results for invasive and *in situ* breast cancers were broadly similar with no significant differences.

## Discussion

Increased breast cancer risk was associated with an earlier thelarche, earlier menarche, earlier regular periods, a longer time between thelarche and menarche, or a shorter time between menarche and the onset of regular periods. Findings were generally similar when premenopausal and postmenopausal breast cancer were analysed separately and in sensitivity analyses.

There is evidence that breast cancer aetiology comprises several pathways, some of which have their origins early in life [14,15]. It has been postulated that a larger number of undifferentiated cells in the breast might increase breast cancer risk [14,15], because undifferentiated cells appear to be more liable to undergo malignant transformation than differentiated cells, based on epidemiological [14] and rat [16] studies. Another postulated pathway relates to hormones, such as oestrogen, that increase cell proliferation, thereby increasing the chance of harmful mutations occurring during DNA replication. These pathways suggest that the timing of puberty could potentially affect breast cancer risk because the number of undifferentiated breast cells increases rapidly during puberty [16] and it is a time of substantial changes in the hormonal environment [17].

Thelarche marks the start of pubertal breast growth after a period of relatively few changes during childhood [16]. Our findings provided evidence that earlier thelarche may be associated with a higher risk of breast cancer. We can find no previous data on the effects of age at thelarche on breast cancer risk [18]. There are plausible biological mechanisms by which early thelarche might increase risk. At puberty, breast ducts begin to grow and divide, and the ends of the ducts form terminal end buds [16]. The breast comprises predominantly undifferentiated, proliferating cells until the first full-term pregnancy and lactation, at which point a large proportion of breast cells differentiate fully [16]. Therefore, an earlier thelarche might suggest a longer time during which the breast comprises a high number of undifferentiated cells and thus has a higher risk of harmful mutations developing [14,15], particularly among women who have the same age at first full-term pregnancy.

Menarche also marks a key stage of breast development. At menarche, the rate at which breast ducts grow and divide increases [19]. Early menarche is a well-established breast cancer risk factor [6,20,21] with our estimated HRs broadly consistent with those seen in the literature [6]. The effect of early menarche might be due to oestrogens, because they increase breast cell proliferation [22] and have been related to breast cancer risk [23], to a larger number of undifferentiated cells due to increased mammary gland size after menarche, and/or to a potentially longer time when the breast is largely undifferentiated among women with a similar age at first pregnancy.

A longer duration from thelarche to menarche also appeared to increase breast cancer risk independently of the effect of age at thelarche in regression analyses when age at thelarche was included as a potential confounder. This does not seem to have been investigated previously. However, the period between thelarche and menarche is when breast duct development largely happens, so if cells are more susceptible when in the ductal growth phase, one might expect such an association with duration from thelarche to menarche. In the context of radiation-induced breast cancer, at least, there is clear evidence that the breast is more susceptible to a carcinogenic exposure around the time of puberty than at other times [24].

Some [7,8], but not all [25], studies have found a higher risk of breast cancer among women whose menses became regular soon after menarche compared with those whose menses took longer to regulate or never became regular. We found evidence to support this. It has been suggested that women who have irregular cycles have a reduced breast cancer risk because levels of ovarian hormones are highest during the luteal phase of the menstrual cycle and women with irregular cycles would spend relatively less time in this phase than women who have regular cycles [7]. This would suggest that age at regular cycles would be associated with breast cancer risk, not just time to regular cycles, which was the case in our study in most, but not all, analyses.

Existing evidence suggests that rapid growth in childhood and adolescence may increase breast cancer risk [26,27]. In the BGS, we did not find this in analyses in which we estimated height velocity between ages 7 and 11 years by comparing categories of relative height at these ages. Although these were comparative data based on recall, some BGS participants (*N* = 199) reported their absolute height at these ages because they or their parents had kept records. Based on these recorded data, the average increase in height from age 7 to 11 was 26.8 cm in the growth spurt group and 23.4 cm among participants who were not classed as having had a growth spurt (*P* <0.01 for difference), suggesting that our growth spurt measure was a reasonable proxy.

It is also of interest whether the timing of the growth spurt affects breast cancer risk. The age at peak growth is one year earlier than menarche on average [28] and was associated with increased breast cancer risk in a large Danish study [20]. Age at which adult height was reached is correlated with age at peak growth [29,30] and, unlike age at peak growth, it can be ascertained by recall. Generally, studies have found that older age at attained height, and thus presumptively older age at peak growth, is associated with a lower breast cancer risk [29,31], but no such association was present in the Nurses’ Health Study II [30]. Our data gave limited support: although no association was found in participants overall, there was an association among women aged 60 years or younger at recruitment, whose recall might be better.

The timing of pubertal events is likely to be correlated to some extent, which may partly explain our findings and the fact that many of the effect sizes were similar across pubertal events. However, Table 4 shows that the correlation between event timing was low in most instances. Interestingly, the duration between thelarche and menarche was not correlated with age at thelarche or age at menarche, but was highly correlated with age at regular periods. Likewise, age at thelarche was highly correlated with time between menarche and regular periods, but not with age at menarche or age at regular periods. These high correlations suggest that these risk factors may be acting through the same pathway as each other or one may be a marker for the other.

**Table 4:** Correlations between timing of pubertal variables

	**Thelarche**	**Menarche**	**Regular periods**	**Reached adult height**	**Thelarche to menarche**	**Thelarche to regular periods**	**Menarche to regular periods**
Menarche	0.02						
Regular periods	0.02	0.58					
Reached adult height	0.00	0.34	0.22				
Thelarche to menarche	0.00	−0.01	0.81	0.03			
Thelarche to regular periods	−1.00	−0.02	−0.01	−0.00	−0.00		
Menarche to regular periods	−1.00	−0.02	−0.01	−0.00	0.01	1.00	
Menarche to adult height	−0.01	−0.36	−0.19	0.75	0.03	0.01	0.01

These analyses included comprehensive adjustment for potential confounders. While benign breast disease is an established risk factor for breast cancer, we did not adjust for it as it is unlikely to be a confounder of the relationship between puberty variables and breast cancer. It is possible that benign breast disease is on the same causal pathway and is a mediator of the relationship between pubertal variables and breast cancer risk, though evidence linking pubertal factors to benign breast disease is lacking.

The pubertal variables that we analysed were reported retrospectively; therefore their accuracy should be considered. Whereas menarche is a well-defined single event that women can recall with reasonable accuracy (for example, 90% accurately recalled the age within a year in one study [2]), the onset of breast development, regular menses and peak growth cannot be ascertained by a single event and, hence, recall is likely to be less accurate. There is some evidence of this inaccuracy for regular menses [2,32], but the validity of recalled age at thelarche does not appear to have been studied previously and we had to rely on proxies for age at peak growth. However, the timing of pubertal stages was reported at baseline before breast cancer diagnosis and so it is unlikely that recall of the pubertal stages would be biased with respect to a subsequent breast cancer diagnosis. Thus, the likely effect of inaccurate recall would have been to attenuate any true associations.

Another limitation of the present analyses is that the average follow-up was only four years. Despite this, more than 1,000 breast cancers had occurred due to the size of the cohort. In addition to the large sample size, the other strengths of this study were its prospective nature, and that it examined several potential breast cancer risk factors for which there has been no or little existing research.

While the only inclusion criteria for women to join the BGS were that they must be aged at least 16 years and living in the UK, there are indications that the study participants are on average a somewhat high-risk population - they tend to be of higher socioeconomic status and more often to have a family history of breast cancer than the UK general population [10]. There seems no reason, however, why this should have affected the associations, as opposed to the distribution of risk factors, reported here.

## Conclusions

In conclusion, the novel findings of this study are that earlier thelarche and a longer time between thelarche and menarche might increase breast cancer risk, as well as the already-investigated effects of early menarche, regular periods and the time between the two. This suggests that breast duct development may be a period of raised susceptibility to breast carcinogenesis, and that the association between puberty and breast cancer is complex and not best represented by a single event. Further research on the effects of pubertal stages other than age at menarche is needed to elucidate these relationships.

## Abbreviations

BGS: Breakthrough generations study; HRT: Hormone replacement therapy.

## Competing interests

All authors declare that they have no competing interests.

## Authors' contributions

AJS and AA are Principal Investigators of the Breakthrough Generations Study and conceived the study. All authors have contributed towards the design and data collection of the Breakthrough Generations Study. DHB conceived these analyses and planned and performed them with input from MJS, MEJ, EM and JG. DHB wrote the first draft of the manuscript and all authors contributed to data interpretation and revisions of the manuscript. All authors have approved the final version of the manuscript.

## Supplementary Material

Additional file 1: Table S1 Risk of invasive or *in situ* breast cancer in relation to pubertal variables, by menopausal status at breast cancer incidence. **Table S2.** Risk of invasive or *in situ* breast cancer in relation to duration between pubertal events, by menopausal status at breast cancer incidence. **Table S3.** Sensitivity analyses of risk of invasive or *in situ* breast cancer in relation to pubertal variables. **Table S4.** Sensitivity analyses of risk of invasive or *in situ* breast cancer in relation to duration between pubertal events. Click here for file
