# Correction to: Timing of pubertal stages and breast cancer risk: the Breakthrough Generations Study

**DOI:** 10.1186/s13058-020-1257-2

**Published:** 2020-02-11

**Authors:** Danielle H. Bodicoat, Minouk J. Schoemaker, Michael E. Jones, Emily McFadden, James Griffin, Alan Ashworth, Anthony J. Swerdlow

**Affiliations:** 1 Division of Genetics and Epidemiology, Institute of Cancer Research, Sutton, UK; 2 Diabetes Research Centre, University of Leicester, Leicester, UK; 3 Department of Primary Care Health Sciences, Oxford University, Oxford, UK; 4 Breakthrough Breast Cancer Research Centre and Division of Molecular Pathology, Institute of Cancer Research, London, UK; 5 Division of Breast Cancer Research, Institute of Cancer Research, Sutton, UK; 6 Diabetes Research Centre, University of Leicester, Leicester Diabetes Centre, Leicester General Hospital, Gwendolen Road, Leicester, Leicestershire LE5 4PW UK

**Correction to: Breast Cancer Res**

**https://doi.org/10.1186/bcr3613**

As a consequence of responding to colleagues who asked about the publication of the original article [1], the authors have determined that the data published in Table 4 of the paper are incorrect. A number of moderate to strong correlations were identified in the corrected data that were not identified in the original manuscript.

The corrected table is shown below. These corrections affect the following sentences in the Discussion, but do not affect the remainder of the findings in the paper, or the summary of data in the Abstract.

The following sentences in the Discussion “Table 4 *shows that the correlation between event timing was low in most instances. Interestingly, the duration between thelarche and menarche was not correlated with age at thelarche or age at menarche, but was highly correlated with age at regular periods, but not with age at menarche or age at regular periods. These high correlations suggest that these risk factors may be acting through the same pathway as each other or one may be a marker for the other”* should be replaced by

“Table 4*shows that the duration between thelarche and menarche was only modestly correlated (− 0.37 and 0.39) with age at thelarche or age at menarche, and was poorly correlated with age at regular periods (0.19). Strong (> 0.70) correlations were observed, however, for age at regular periods with respect to durations to this age from thelarche and menarche, for the duration between menarche and reaching adult height with age at reaching adult height, between the duration from menarche to regular periods and from thelarche to regular periods, and between ages at menarche and thelarche”*

**Table 4:** Pearson’s correlations between timing pubertal variables

	Age at thelarche	Age at menarche	Age at first regular periods	Age reached adult height	Years between thelarche and menarche	Years between thelarche and first regular periods	Years between menarche and first regular periods
Age at menarche	0.71						
Age at regular periods	0.30	0.44					
Age reached adult height	0.33	0.33	0.15				
Years between thelarche and menarche	−0.37	0.39	0.19	0.02			
Years between thelarche and first regular periods	−0.19	0.18	0.80	0.03	0.49		
Years between menarche and first regular periods	−0.004	− 0.01	0.79	0.02	−0.007	0.87	
Years between menarche and reaching adult height	−0.19	−0.38	− 0.17	0.75	− 0.25	−0.09	0.03
