# Supplementary material for: Timing of pubertal stages and breast cancer risk: the Breakthrough Generations Study
Source: Breast Cancer Res. 2014 Feb 4;16(1):R18. doi: 10.1186/bcr3613 (PMC3978643; doi:10.1186/bcr3613)
Supplement: Additional file 1: Table S1 — Risk of invasive or in situ breast cancer in relation to pubertal variables, by menopausal status at breast cancer incidence. Table S2. Risk of invasive or in situ breast cancer in relation to duration between pubertal events, by menopausal status at breast cancer incidence. Table S3. Sensitivity analyses of risk of invasive or in situ breast cancer in relation to pubertal variables. Table S4. Sensitivity analyses of risk of invasive or in situ breast cancer in relation to duration between pubertal events. [file bcr3613-S1.pdf]

Supplementary Table 1. Risk of Invasive or *In Situ* Breast Cancer in Relation to Pubertal Variables, by Menopausal Status at Breast Cancer Incidence.

	Premenopausal			Postmenopausal		
Pubertal variable	*N* cases/ person-years	Adjusted[a] HR (95% CI)		*N* cases/ person-years	Adjusted[a] HR (95% CI)	
Age at thelarche, *years*						
≤10	50/31646	1.28 (0.92, 1.78)		101/21657	1.21 (0.97, 1.52)	
11-12	129/93573	Referent		314/79815	Referent	
≥13	63/55694	0.76 (0.56, 1.03)		206/62843	0.82 (0.69, 0.98)	*
HR for trend		0.77 (0.64, 0.93)	**		0.82 (0.73, 0.92)	**
Age at menarche, *years*						
≤12	125/85966	1.18 (0.91, 1.53)		324/82511	1.02 (0.87, 1.19)	
13-14	111/84229	Referent		333/84504	Referent	
≥15	17/17924	0.67 (0.40, 1.12)		66/20184	0.82 (0.63, 1.07)	
HR for trend		0.80 (0.65, 0.97)	*		0.93 (0.83, 1.04)	
Age at regular periods, *years*						
≤12	58/38570	1.08 (0.77, 1.53)		154/40880	0.96 (0.78, 1.19)	
13-14	76/50827	Referent		216/54487	Referent	
≥15	23/23091	0.66 (0.41, 1.05)		81/26374	0.77 (0.60, 0.99)	*
Never had regular periods	17/13697	1.13 (0.66, 1.91)		56/11884	1.18 (0.88, 1.58)	
HR for trend[b]		0.81 (0.65, 1.01)			0.91 (0.80, 1.03)	
Age reached adult height, *years*						
≤14	52/35822	1.14 (0.79, 1.64)		146/36597	1.31 (1.03, 1.65)	*
15-16	67/54299	Referent		141/44336	Referent	
≥17	40/38938	0.87 (0.59, 1.29)		108/28928	1.17 (0.91, 1.50)	
HR for trend		0.88 (0.71, 1.08)			0.93 (0.82, 1.06)	
Growth spurt between ages 7 and 11 years						
No	249/190051	Referent		698/182031	Referent	
Yes	29/17698	1.26 (0.86, 1.86)		69/16561	1.07 (0.84, 1.37)	

Abbreviations: CI, Confidence interval; HR, Hazard ratio.

* P<0.05; ** P<0.01; *** P<0.001.

[a] Adjusted for attained age, family history of breast cancer in a first-degree relative, adult height, age at first full-term pregnancy (with a term for nulliparous women) and hormone replacement therapy status.

[b] Test for trend excludes those who had never had a regular period.

Supplementary Table 2. Risk of Invasive or *In Situ* Breast Cancer in Relation to Duration Between Pubertal Events, by Menopausal Status at Breast Cancer Incidence.

	Premenopausal			Postmenopausal		
Pubertal variable	*N* cases/ person-years	Adjusted[a] HR (95% CI)		*N* cases/ person-years	Adjusted[a] HR (95% CI)	
Thelarche to menarche, *years*						
<0	20/12926	1.37 (0.83, 2.26)		37/14454	0.72 (0.51, 1.03)	
0	64/52498	Referent		207/58670	Referent	
1	79/60246	1.12 (0.80, 1.55)		220/54721	1.15 (0.95, 1.39)	
≥2	56/36910	1.43 (1.00, 2.04)		99/23391	1.21 (0.95, 1.54)	
HR for trend		1.09 (0.94, 1.27)			1.15 (1.04, 1.27)	**
Thelarche to regular periods, *years*						
<0	9/4284	1.35 (0.65, 2.82)		15/5450	0.74 (0.43, 1.28)	
0	33/20303	Referent		94/25448	Referent	
1	45/33407	0.80 (0.51, 1.26)		132/37188	0.95 (0.73, 1.24)	
≥2	58/43573	0.87 (0.57, 1.33)		129/36576	0.95 (0.73, 1.24)	
Never became regular	17/13697	1.02 (0.57, 1.84)		56/11884	1.24 (0.89, 1.73)	
HR for trend[b]		0.91 (0.76, 1.09)			1.01 (0.90, 1.14)	
Menarche to regular periods, *years*						
0	99/63264	Referent		277/69438	Referent	
1	45/32900	0.86 (0.60, 1.23)		133/37849	0.87 (0.71, 1.07)	
≥2	13/16324	0.55 (0.31, 0.98)	*	41/14454	0.71 (0.51, 0.99)	*
Never became regular	17/13697	1.06 (0.63, 1.78)		56/11884	1.16 (0.87, 1.55)	
HR for trend[b]		0.78 (0.62, 0.99)	*		0.86 (0.74, 0.99)	*
Menarche to adult height, *years*						
<0	4/2694	1.07 (0.38, 3.03)		10/3177	0.85 (0.44, 1.63)	
0-1	32/23843	Referent		98/26649	Referent	
2-3	58/45246	1.06 (0.69, 1.63)		131/37666	0.93 (0.72, 1.21)	
≥4	45/44116	0.89 (0.56, 1.40)		120/33443	0.97 (0.74, 1.26)	
HR for trend		0.94 (0.77, 1.15)			1.00 (0.88, 1.13)	

Abbreviations: CI, Confidence interval; HR, Hazard ratio.

[*] P<0.05; ** P<0.01; *** P<0.001.

[a] Adjusted for attained age, menopausal status, family history of breast cancer in a first-degree relative, adult height, age at first full-term pregnancy (with a term for nulliparous women) and hormone replacement therapy use.

[b] Test for trend excludes those who had never had a regular period.

Supplementary Table 3. Sensitivity Analyses of Risk of Invasive or *In Situ* Breast Cancer in Relation to Pubertal Variables.

	Age <60 years at baseline (n=85122)		Excluding younger relatives (n=73560)		Invasive breast cancer only as outcome (n=104931)		*In situ* breast cancer only as outcome (n=92209)	
Pubertal variable	Adjusted[a] HR (95% CI)		Adjusted[a] HR (95% CI)		Adjusted[a] HR (95% CI)		Adjusted[a] HR (95% CI)	
Age at thelarche, *years*								
≤10	1.30 (1.06, 1.60)	*	1.26 (1.01, 1.57)	*	1.25 (1.02, 1.52)	*	1.14 (0.67, 1.94)	
11-12	Referent		Referent		Referent		Referent	
≥13	0.82 (0.69, 0.99)	*	0.79 (0.66, 0.95)	*	0.83 (0.70, 0.97)	*	0.62 (0.39, 1.00)	*
HR for trend	0.80 (0.71, 0.90)	***	0.80 (0.71, 0.90)	***	0.82 (0.74, 0.91)	***	0.73 (0.55, 0.97)	*
Age at menarche, *years*								
≤12	1.06 (0.91, 1.24)		1.05 (0.89, 1.23)		1.06 (0.92, 1.22)		1.04 (0.72, 1.50)	
13-14	Referent		Referent		Referent		Referent	
≥15	0.75 (0.56, 1.00)	*	0.66 (0.49, 0.90)	**	0.88 (0.69, 1.12)		0.15 (0.04, 0.61)	**
HR for trend	0.88 (0.78, 0.99)	*	0.86 (0.77, 0.97)	*	0.92 (0.83, 1.02)		0.70 (0.52, 0.95)	*
Age at regular periods, *years*								
≤12	1.03 (0.83, 1.27)		0.95 (0.77, 1.18)		1.06 (0.88, 1.28)		0.60 (0.35, 1.02)	
13-14	Referent		Referent		Referent		Referent	
≥15	0.69 (0.52, 0.91)	**	0.68 (0.52, 0.90)	**	0.77 (0.60, 0.98)	*	0.53 (0.27, 1.02)	
Never had regular periods	1.30 (0.97, 1.75)		1.09 (0.79, 1.49)		1.24 (0.94, 1.62)		0.75 (0.34, 1.66)	
HR for trend[b]	0.84 (0.74, 0.96)	*	0.88 (0.77, 1.00)		0.86 (0.77, 0.97)	*	1.01 (0.74, 1.38)	
Age reached adult height, *years*								
≤14	1.24 (0.99, 1.55)		1.41 (1.11, 1.80)	**	1.30 (1.06, 1.60)	*	0.90 (0.49, 1.66)	
15-16	Referent		Referent		Referent		Referent	
≥17	0.93 (0.72, 1.19)		1.16 (0.89, 1.50)		1.06 (0.85, 1.33)		1.11 (0.61, 2.01)	
HR for trend	0.86 (0.76, 0.98)	*	0.89 (0.78, 1.02)		0.89 (0.79, 1.01)		1.11 (0.80, 1.54)	
Growth spurt between ages 7 and 11 years								

No	Referent	Referent	Referent	Referent
Yes	1.24 (0.97, 1.57)	1.19 (0.93, 1.52)	1.20 (0.97, 1.49)	0.56 (0.25, 1.27)

Abbreviations: CI, Confidence interval; HR, Hazard ratio.

[*] P<0.05; ** P<0.01; *** P<0.001.

[a] Adjusted for attained age, family history of breast cancer in a first-degree relative, adult height, age at first full-term pregnancy (with a term for nulliparous women) and hormone replacement therapy status.

[b] Test for trend excludes those who had never had a regular period.

Supplementary Table 4. Sensitivity Analyses of Risk of Invasive or *In Situ* Breast Cancer in Relation to Duration Between Pubertal Events.

	Age <60 years at baseline (n=85122)		Excluding younger relatives (n=73560)		Invasive breast cancer only (n=104931)		*In situ* breast cancer only as outcome (n=104931)	
Pubertal variable	Adjusted[a] HR (95% CI)		Adjusted[a] HR (95% CI)		Adjusted[a] HR (95% CI)		Adjusted[a] HR (95% CI)	
Thelarche to menarche, *years*								
<0	0.86 (0.62, 1.20)		1.02 (0.74, 1.41)		0.93 (0.69, 1.25)		0.47 (0.17, 1.33)	
0	Referent		Referent		Referent		Referent	
1	1.09 (0.90, 1.32)		1.17 (0.95, 1.43)		1.13 (0.95, 1.35)		1.19 (0.75, 1.88)	
≥2	1.13 (0.90, 1.43)		1.33 (1.05, 1.70)	*	1.32 (1.07, 1.63)	**	0.93 (0.50, 1.74)	
HR for trend	1.08 (0.99, 1.19)		1.12 (1.02, 1.24)	*	1.13 (1.04, 1.24)	**	1.13 (0.89, 1.43)	
Thelarche to regular periods, *years*								
<0	0.89 (0.53, 1.50)		1.02 (0.63, 1.63)		0.95 (0.61, 1.49)		0.38 (0.05, 2.96)	
0	Referent		Referent		Referent		Referent	
1	0.93 (0.71, 1.22)		0.80 (0.61, 1.06)		0.90 (0.71, 1.15)		1.05 (0.51, 2.17)	
≥2	0.84 (0.64, 1.09)		0.85 (0.65, 1.12)		0.86 (0.67, 1.09)		1.63 (0.83, 3.20)	
Never became regular	1.24 (0.89, 1.73)		1.08 (0.76, 1.53)		1.18 (0.87, 1.59)		1.26 (0.49, 3.19)	
HR for trend[b]	0.94 (0.84, 1.05)		0.92 (0.82, 1.04)		0.94 (0.85, 1.04)		1.38 (1.01, 1.87)	*
Menarche to regular periods, *years*								
0	Referent		Referent		Referent		Referent	
1	0.77 (0.62, 0.95)	*	0.89 (0.72, 1.10)		0.88 (0.72, 1.06)		0.82 (0.48, 1.41)	
≥2	0.63 (0.45, 0.87)	**	0.61 (0.43, 0.88)	**	0.57 (0.41, 0.79)	**	1.41 (0.76, 2.63)	
Never became regular	1.21 (0.91, 1.62)		1.09 (0.80, 1.49)		1.15 (0.88, 1.50)		0.97 (0.44, 2.16)	
HR for trend[b]	0.78 (0.68, 0.91)	**	0.82 (0.71, 0.95)	**	0.80 (0.70, 0.91)	**	1.11 (0.81, 1.51)	
Menarche to adult height, *years*								
<0	0.89 (0.46, 1.70)		1.07 (0.56, 2.06)		0.90 (0.51, 1.59)		1.00 (0.12, 8.00)	

0-1	Referent	Referent	Referent	Referent
2-3	0.95 (0.73, 1.24)	1.03 (0.78, 1.36)	0.90 (0.71, 1.13)	2.00 (0.90, 4.46)
≥4	0.90 (0.69, 1.18)	1.00 (0.75, 1.32)	0.87 (0.68, 1.11)	2.02 (0.90, 4.46)
HR for trend	0.96 (0.85, 1.09)	0.99 (0.87, 1.13)	0.95 (0.85, 1.06)	1.32 (0.95, 1.84)

Abbreviations: CI, Confidence interval; HR, Hazard ratio.

[*] P<0.05; ** P<0.01; *** P<0.001.

[a] Adjusted for attained age, menopausal status, family history of breast cancer in a first-degree relative, adult height, age at first full-term pregnancy (with a term for nulliparous women) and HRT use.

[b] Test for trend excludes those who had never had a regular period.
